# Receiving coupons and discounts for nicotine pouches is associated with current use of nicotine pouches among United States adults: Results from the population assessment of tobacco and health (PATH) study wave 7 (2022–2023)

**DOI:** 10.1016/j.dadr.2025.100370

**Published:** 2025-08-06

**Authors:** Juhan Lee, Frank Merenda, Andrea H. Weinberger

**Affiliations:** a Department of Epidemiology and Population Health, Albert Einstein College of Medicine, United States; b Department of Epidemiology and Population Health, Montefiore Einstein Comprehensive Cancer Center, United States; c Ferkauf Graduate School of Psychology, Yeshiva University, United States; d Department of Psychiatry and Behavioral Sciences, Albert Einstein College of Medicine, United States

**Keywords:** Marketing, Nicotine pouches, Epidemiology

## Abstract

**Background:**

The rising use of nicotine pouches has emerged as a public health concern. Increasing use might be driven by exposure to marketing (e.g., discounts, coupons) but little is known about marketing and nicotine pouch use. This study is the first to use United States (US) nationally representative data to examine the relationship between exposure to discounts and coupons for nicotine pouches and nicotine pouch use among US adults.

**Methods:**

Using respondents from the Population Assessment of Tobacco and Health (PATH) Study Wave 7 (2022–2023) dataset (N = 27,712), we examined the association between past 12-month exposure to nicotine pouch discounts/coupons and past-30-day nicotine pouch use, adjusting for sociodemographic, psychological, and behavioral covariates.

**Results:**

Among respondents, 0.3 % (weighted) reported past-12-month receipt of discounts or coupons for nicotine pouches, and 0.8 % reported past-30-day use of nicotine pouches, with a significant bivariate association (p < 0.001). After controlling for covariates, individuals exposed to discounts or coupons for nicotine pouches during the past 12 months were more likely to report past-30-day use of nicotine pouches (adjusted odds ratio=33.85, 95 % confidence interval=19.13, 59.92).

**Conclusions:**

Exposure to discounts/coupons for nicotine pouches was associated with an increased risk of product use in the past 30 days among US adults. Continual surveillance of tobacco marketing strategies and sustained research efforts are necessary to inform tobacco control efforts and cessation strategies related to nicotine pouches.

## Highlights

• Among US adults in 2022–2023, 0.3 % reported past-12-month receipt of discounts or coupons for nicotine pouches.
• Among US adult in 2022–2023, 0.8 % reported past-30-day use of nicotine pouches.
• US adults exposed to discounts or coupons for nicotine pouches were more likely to report use of nicotine pouches.

## 1 Introduction

Nicotine pouches have gained popularity in recent years. Sales of nicotine pouches have drastically increased in the United States (US) from 126 million in 2019–808 million in 2022 (Majmundar et al., 2022a, Liu and Halpern-Felsher, 2024, US Food and Drug Administration,, Felicione et al., 2025). Nicotine pouches are “*small microfiber pouches that contain a powder made of nicotine, flavorings, and other ingredients*,” placed between the lip and gum, where the nicotine is absorbed (Center for Disease Control and Prevention,). The use of nicotine pouches and other nicotine products is a new public health concern given the introduction of a greater number of brands in the US commercial marketplace (e.g., Zyn in 2014, Velo in 2019, On! in 2019), and these brands’ active marketing activities in new media channels reaching wider, younger audiences (e.g., social media) (Felicione et al., 2025, Phan et al., 2024). Several reviews suggest that nicotine pouches might be less harmful than combustible cigarette smoking and moist snuff, (Travis et al., 2024a, Zamarripa et al., 2024) but they still contain nicotine and other chemicals (e.g., formaldehyde) (Kent et al., 2024, Travis et al., 2024b). Thus, caution and continuous surveillance of the use of these tobacco products, along with associated factors that increase the risk of use, are important.

Manufacturers of nicotine pouches demonstrated significant marketing expenditures on various marketing strategies for these products during 2016–2023 (Emery et al., 2023, Ozga et al., 2024, Duan et al., 2024). One common general marketing strategy is providing coupons and discounts for tobacco products (US Department of Health and Human Services, 2025a, US Department of Health and Human Services, 2025b, U.S. Department of Health and Human Services, 2016). Previous studies have found that coupons were commonly included in advertisements for nicotine pouches, and the proportion of ads with coupons or discounts (e.g., percent-off sales) significantly increased from 2022 to 2023 (Czaplicki et al., 2022, Czaplicki et al., 2024, Talbot et al., 2023). However, these studies did not examine whether exposure to these discount and coupon marketing strategies was related to the actual use of nicotine pouches. One recent study found that exposure to nicotine pouch marketing (e.g., retail, internet, print, outdoor ads) was associated with increased levels of ever use of nicotine pouches among US adults with current or former tobacco use (Phan et al., 2024). However, few studies have specifically examined the association between exposure to price discounts and coupons and their use.

This study used national data to examine the association between exposure to discounts and coupons for nicotine pouches and their use among US adults. We hypothesized that receiving discounts and coupons for nicotine pouches would be associated with an increased prevalence of use.

## 2 Methods

### 2.1 Dataset and analytic sample

This study used adult respondent data from the Population Assessment of Tobacco and Health (PATH) Study Wave 7 (2022–2023) public dataset (N = 29,780). The PATH Study is conducted by the National Institute of Drug Abuse and the FDA Center for Tobacco Products. It assesses tobacco use behaviors and related factors, using a multistage, stratified sampling of US civilian, non-institutionalized populations, enabling researchers to calculate US nationally representative estimates (Hyland et al., 2017). The PATH Study Wave 7 was the first wave to assess the use of nicotine pouches. The data collection of the PATH Study was approved by the Westat Institutional Review Board, and the secondary data analysis of the de-identified, publicly available US national dataset is exempt from review board insight.

### 2.2 Measures

As an outcome, we used the “past-30-day use of nicotine pouches” (no, yes) consistent with prior research. ^22^ The assessment for past-30-day nicotine pouch use was “*In the past 30 days, have you used nicotine pouches, even one or two times?*” along with the instructions: “*the next questions ask about small, white pouches that contain nicotine which users place in their mouth. Nicotine pouches are different from other smokeless tobacco products such as snus, dip or chew, because they do not contain any ground tobacco leaf. Common brands include Zyn, on!, or Velo, but there are many others. Please do not include other types of pouches such as snus pouches or smokeless tobacco pouches when answering the following questions. [SHOW GENERIC IMAGE OF NICOTINE POUCHES*]”

As a predictor of interest, we used “past-12-month receiving discounts or coupons for nicotine pouches (no, yes).” This variable was assessed using the item “*In the past 12 months, have you received discounts or coupons for any of the following products? Choose all that apply*.” The response options included “Nicotine pouches (such as Zyn, On!, or Velo).” “Past-12-months” was the only timeframe assessed in the dataset.

As covariates, we included sociodemographics related to tobacco use (US Department of Health and Human Services, 2025b). The covariates were age (18–24 years, 25–44 years, 45–64 years, ≥65 years), sex (female, male), race (White, Black, Other), ethnicity (non-Hispanic, Hispanic), sexual identity (heterosexual, sexual minority [lesbian, gay, bisexual, or other identity]), income level ($50,000 as cut-off). Due to the small outcome event by predictor, we selected covariates conservatively to avoid potential bias of overadjustment and small sample bias (Rassen et al., 2011, Rothman et al., 2008, Peduzzi et al., 1996). We ran a sensitivity analysis with additional psychological and behavioral covariates to avoid under-adjustment bias (described below).

### 2.3 Statistical analysis

We first conducted descriptive statistics of sample characteristics among the total adult respondents and stratified by past-30-day use of nicotine pouches. Using the Rao–Scott adjusted Pearson chi-squared test, we tested the significance of bivariate associations. We then conducted an adjusted binomial logistic regression model on the past-30-day use of nicotine pouches by past-12-month receiving discounts or coupons for nicotine pouches (Peduzzi et al., 1996, Doerken et al., 2019). The model was adjusted for sociodemographics.

We conducted four sensitivity analyses. First, to avoid the potential bias from the small sample size of the outcome event by the primary predictor, we ran the same model using an adjusted robust-error-variance Poisson regression model (i.e., modified Poisson regression model) (Barros and Hirakata, 2003, Zou, 2004, Dai and Leventhal, 2024). Second, since nicotine pouches might be more popular among young adults, (Dai and Leventhal, 2024) we ran the same model only among young adults (18–24 years old; N = 10,310). Third, to avoid potential bias from under-adjustment of potential confounders, we included additional covariates, past-30-day cigarette smoking, e-cigarette use, smokeless tobacco or snus use, other substance use (e.g., alcohol, cannabis, cocaine or crack, misuse of painkillers, sedatives or tranquilizers, stimulants such as methamphetamine or speed, heroin, inhalants or solvents, hallucinogens), and internalizing and externalizing tendencies (assessed with the Global Appraisal of Individual Needs - Short Screener [GAIN-SS]) (Dennis et al., 2007). Fourth, to examine continued use of nicotine pouches related to receiving discounts and coupons, we ran the same model only among adult respondents who reported ever use of nicotine pouches (N = 1408).

We applied the Wave 7 cross-sectional sampling weight for all analyses, and the variance estimation was conducted by Fay (0.3) adjusted balanced repeated replication methods. From a complete-case approach of the logistic regression model, the final analytic sample included N = 27,712. We considered p < 0.05 (two-tailed) statistically significant and followed the Strengthening the Reporting of Observational Studies in Epidemiology (STROBE) guidelines. STATA 18.0 (College Station, TX, USA) was used for all statistical analyses.

## 3 Results

Table 1 shows the sample characteristics among the total analytic sample (N = 29,890) and stratified by past-30-day use of nicotine pouches. Among the total adult respondents, 11.7 % (weighted) were young adults aged 18–24 years, 51.5 % were women, 12.4 % were Black, 16.9 % were Hispanic, 9.6 % were sexual minority adults, and 44.5 % made less than $50,000 annual income. Of the total adult respondents, 0.8 % (weighted) reported past-30-day use of nicotine pouches (outcome), and 0.3 % reported past-12-month receiving discounts or coupons for nicotine pouches (predictor of interest), with a significant bivariate association (p < 0.001).

**Table 1:** Sample characteristics among overall adult respondents and stratified by past-30-day use of nicotine pouches.

		**Past-30-day use of nicotine pouches**		
	**Total**	**No**	**Yes**	**p-value**^a^
		**Unweighted n = 29,379**	**Unweighted n = 375**	
		**Weighted %=99.2**	**Weighted %=0.8**	
**Past−12-month receiving discounts or coupons of nicotine pouches**				<0.001
None	29,501 (99.7)	29,143 (99.3)	335 (0.7)	
Any	134 (0.3)	98 (77.1)	36 (22.9)	
**Age, years**				<0.001
18–24	10,310 (11.7)	10,160 (98.5)	141 (1.5)	
25–44	10,496 (34.2)	10,314 (98.6)	175 (1.4)	
45–64	5832 (32.5)	5772 (99.5)	56 (0.5)	
≥ 65	3140 (21.6)	3131 (99.9)	3 (0.1)	
**Sex**				<0.001
Female	15,695 (51.5)	15,613 (99.7)	69 (0.3)	
Male	14,085 (48.5)	13,766 (98.6)	306 (1.4)	
**Race**				<0.001
White	20,811 (74.6)	20,469 (99.0)	322 (1.0)	
Black	5243 (12.4)	5223 (99.7)	18 (0.3)	
Other	3726 (13.1)	3687 (99.4)	35 (0.6)	
**Ethnicity**				0.001
Non-Hispanic	22,949 (83.1)	22,592 (99.1)	338 (0.9)	
Hispanic	6831 (16.9)	6787 (99.6)	37 (0.4)	
**Sexual identity**				0.877
Heterosexual	24,784 (90.4)	24,438 (99.2)	329 (0.8)	
Lesbian, gay, bisexual, or other identity	4596 (9.6)	4548 (99.2)	42 (0.8)	
**Annual household income level**				0.014
Less than $50,000	14,495 (44.5)	14,340 (99.3)	144 (0.7)	
$50,000 or more	13,541 (55.5)	13,309 (99.0)	222 (1.0)	

a p-values were tested using Rao-Scott adjusted Pearson chi-squared tests

Table 2 shows the results of the adjusted binomial logistic regression model on past-30-day use of nicotine pouches. After controlling sociodemographic factors, past-12-month receiving discounts or coupons for nicotine pouches (vs. no) was significantly associated with past-30-day use of nicotine pouches (adjusted odds ratio [AOR]=33.85, 95 % confidence interval [CI]=19.13, 59.92). Other factors associated with past-30-day use of nicotine pouches were age (e.g., older age groups were less likely to report past-30-day use than younger age groups), male sex (vs. female; AOR=4.97, 95 % CI=3.42, 7.23), Black race (vs. White; AOR=0.26, 95 % CI=0.13, 0.51), and Hispanic ethnicity (vs. non-Hispanic; AOR=0.37, 95 % CI=0.21, 0.65).

**Table 2:** Results of adjusted binomial logistic regression model on past-30-day use of nicotine pouches, among total adult respondents from PATH Study Wave 7 (2022–2023).

	**Outcome: Past-30-day use of nicotine pouches**	
	**Adjusted Odds Ratio (95 % CI)**	**p-value**
**Past−12-month receiving discounts or coupons of nicotine pouches**		
None	Reference	
Any	**33.85 (19.13, 59.92)**	**< 0.001**
**Age, years**		
18–24	Reference	
25–44	0.76 (0.58, 0.99)	0.041
45–64	0.29 (0.19, 0.42)	< 0.001
≥ 65	0.03 (0.01, 0.12)	< 0.001
**Sex**		
Female	Reference	
Male	4.97 (3.42, 7.23)	< 0.001
**Race**		
White	Reference	
Black	0.26 (0.13, 0.51)	< 0.001
Other	0.47 (0.29, 0.76)	0.002
**Ethnicity**		
Non-Hispanic	Reference	
Hispanic	0.37 (0.21, 0.65)	0.001
**Sexual identity**		
Heterosexual	Reference	
Lesbian, gay, bisexual, or other identity	0.90 (0.61, 1.34)	0.608
**Annual household income level**		
Less than $50,000	Reference	
$50,000 or more	1.00 (0.76, 1.33)	0.983

CI=Confidence Interval

With regard to sensitivity analyses, a similar relationship between past-12-month receiving discounts or coupons for nicotine pouches and past-30-day nicotine pouch use was observed in the adjusted modified Poisson regression model (adjusted prevalence ratio=20.73, 95 % CI=14.31, 30.04) Supplemental Table 1). The association was also significant when limiting the sample to young adults ages 18–24 (AOR=21.87, 95 % CI=7.34, 65.14; Supplemental Table 2), after adjusting for additional psychological and behavioral confounders (AOR=29.32, 95 % CI=14.35, 59.92; Supplemental Table 3), and when examining continued use among adults who reported ever use of nicotine pouches (AOR=5.94, 95 % CI=2.82, 12.51; Supplemental Table 4).

## 4 Discussion

Past-12-month receiving discounts and coupons for nicotine pouches was associated with an increased risk for nicotine pouches use in the past 30 days. This finding was replicated in several sensitivity analyses suggesting robustness (Thabane et al., 2013). Our study is the first to extend previous work relating discounts and coupons for other tobacco products (e.g., cigarettes) to increased levels of tobacco product use, (Liber et al., 2022) to show a similar relationship for nicotine pouches. As of 2025, providing tobacco product discounts and coupons is not federally prohibited (though tobacco product discounts and coupons are prohibited in Providence, Rhode Island, the state of New York, and the state of New Jersey) (Public Health Law Center, nd). As nicotine pouches are available in the marketplace, these results suggest that close monitoring and restriction on their marketing strategies, especially discounts and coupons should be considered.

With regard to other factors associated with nicotine pouch use, we found increased risk for young adults and men and lower risk for individuals who identified as Black race and Hispanic ethnicity. Increased risk for young adults is consistent with previous findings, (Center for Disease Control and Prevention, nd, Palmer et al., 2025, Dai and Leventhal, 2024) and potential reasons might be advertisements displaying themes appealing to young adults (e.g., freedom, innovation, modernity, featuring bars or nightclubs) and the use of young adult models (Duan et al., 2024, Ling et al., 2023). Further, nicotine pouches have been proactively promoted on social media (e.g., “zynfluencers” [social media influencers promoting Zyn]), frequented by young adults (Dobbs et al., 2024).

We also found that males might be at increased risk (Palmer et al., 2025, Jongenelis et al., 2024, Tosakoon et al., 2023). Potential reasons might be male-targeted marketing such as featuring masculinity and male models in the advertisements and placing advertisements in men’s journals (Ozga et al., 2024, Duan et al., 2024). When we controlled additional psychological and behavioral covariates, we found the use of other tobacco product use such as e-cigarettes and smokeless tobacco or snus to be risk factors, also consistent with previous findings (Palmer et al., 2025, Dai and Leventhal, 2024, Jongenelis et al., 2024, Vogel et al., 2022, Patel et al., 2023, Sparrock et al., 2023, Han et al., 2025). These findings might be driven by frequent cross-product promotion by tobacco brands, (Talbot et al., 2023) a motivation to use nicotine pouches to help when quitting other tobacco products, (Dai and Leventhal, 2024, Hrywna et al., 2023) and the use of nicotine pouches when other tobacco product use is not permitted (Han et al., 2025).

In contrast, Black (vs. White) and Hispanic (vs. non-Hispanic) individuals were less likely to report past-30-day use of nicotine pouches, consistent with some previous findings (Dai and Leventhal, 2024, Tosakoon et al., 2023, Patel et al., 2023). However, a study by Phan et al. using a nationally representative sample of US adults with current and former commercial tobacco use found that certain nicotine pouch marketing strategies (e.g., internet/social media ads, direct mail/email marketing) might be significantly associated with actual use among Black adults, even though these individuals were not disproportionately exposed to such marketing (Phan et al., 2024). Continuous surveillance on marketing strategies and usage patterns of nicotine pouches among Hispanic and Black individuals is necessary.

Limitations should be noted. First, this study relied on self-report, which might pose self-report biases. Second, data were cross-sectional so we could not establish a causal relationship between receiving discounts and coupons and use behaviors. Third, we could not characterize nicotine pouches by factors such as flavor use and nicotine concentration as this information was lacking in the dataset (Shaikh et al., 2023, Majmundar et al., 2022b). Fourth, unmeasured confounders are possible, such as harm perceptions, exposure to nicotine pouch advertisements in other channels, and peer use (Phan et al., 2024, Tosakoon et al., 2023, Gaiha et al., 2023, Brierley et al., 2025). Fifth, this study was unable to examine the differences in the association between exposure to coupons and discounts with nicotine pouch use across different sociodemographic groups due to the relatively small sample size of individuals using nicotine pouches in the PATH Study. Future studies with larger samples of individuals with nicotine pouch use should examine differences these relationships by sociodemographic factors to inform future tobacco control policies and interventions.

## 5 Conclusions

Receiving discounts and coupons for nicotine pouches was associated with increased risk for nicotine pouch use. Even though nicotine pouches might be less harmful than other tobacco products, they still have negative health consequences such as exposure to nicotine and other chemicals (Zamarripa et al., 2024, Travis et al., 2024b). While accumulating evidence, continuously monitoring use and marketing strategies is important to inform prevention and cessation strategies and tobacco control efforts.

## CRediT authorship contribution statement

**Juhan Lee:** Writing – review & editing, Writing – original draft, Investigation, Formal analysis, Conceptualization. **Frank Merenda:** Writing – review & editing, Investigation. **Andrea H. Weinberger:** Writing – review & editing, Investigation.

## Funding

None

## Declaration of Competing Interest

The authors declare that they have no known competing financial interests or personal relationships that could have appeared to influence the work reported in this paper.

## Data Availability

The Population Assessment of Tobacco and Health (PATH) Study is deidentified and publicly available at: https://pathstudy.nih.gov/ The statistical code for this secondary data analysis is available upon request.
