# Supplementary material for: Receiving coupons and discounts for nicotine pouches is associated with current use of nicotine pouches among United States adults: Results from the population assessment of tobacco and health (PATH) study wave 7 (2022–2023)
Source: Drug Alcohol Depend Rep. 2025 Aug 6;16:100370. doi: 10.1016/j.dadr.2025.100370 (PMC12356447; doi:10.1016/j.dadr.2025.100370)
Supplement: Supplementary file 1 — Supplementary material [file mmc1.docx]

**Table 1. Sample characteristics among overall adult respondents and stratified by past-30-day use of nicotine pouches**

|  |  | **Past-30-day use of nicotine pouches** | | |
| --- | --- | --- | --- | --- |
|  | **Total** | **No** | **Yes** | **p-value ^a^** |
|  |  | **Unweighted n=29,379** | **Unweighted n=375** |  |
|  |  | **Weighted %=99.2** | **Weighted %=0.8** |  |
| **Past-12-month receiving discounts or coupons of nicotine pouches** |  |  |  | <0.001 |
| None | 29,501 (99.7) | 29,143 (99.3) | 335 (0.7) |  |
| Any | 134 (0.3) | 98 (77.1) | 36 (22.9) |  |
| **Age, years** |  |  |  | <0.001 |
| 18-24 | 10,310 (11.7) | 10,160 (98.5) | 141 (1.5) |  |
| 25-44 | 10,496 (34.2) | 10,314 (98.6) | 175 (1.4) |  |
| 45-64 | 5,832 (32.5) | 5,772 (99.5) | 56 (0.5) |  |
| ≥65 | 3,140 (21.6) | 3,131 (99.9) | 3 (0.1) |  |
| **Sex** |  |  |  | <0.001 |
| Female | 15,695 (51.5) | 15,613 (99.7) | 69 (0.3) |  |
| Male | 14,085 (48.5) | 13,766 (98.6) | 306 (1.4) |  |
| **Race** |  |  |  | <0.001 |
| White | 20,811 (74.6) | 20,469 (99.0) | 322 (1.0) |  |
| Black | 5,243 (12.4) | 5,223 (99.7) | 18 (0.3) |  |
| Other | 3,726 (13.1) | 3,687 (99.4) | 35 (0.6) |  |
| **Ethnicity** |  |  |  | 0.001 |
| Non-Hispanic | 22,949 (83.1) | 22,592 (99.1) | 338 (0.9) |  |
| Hispanic | 6,831 (16.9) | 6,787 (99.6) | 37 (0.4) |  |
| **Sexual identity** |  |  |  | 0.877 |
| Heterosexual | 24,784 (90.4) | 24,438 (99.2) | 329 (0.8) |  |
| Lesbian, gay, bisexual, or other identity | 4,596 (9.6) | 4,548 (99.2) | 42 (0.8) |  |
| **Annual household income level** |  |  |  | 0.014 |
| Less than $50,000 | 14,495 (44.5) | 14,340 (99.3) | 144 (0.7) |  |
| $50,000 or more | 13,541 (55.5) | 13,309 (99.0) | 222 (1.0) |  |

^a^ p-values were tested using Rao-Scott adjusted Pearson chi-squared tests

**Table 2. Results of adjusted binomial logistic regression model on past-30-day use of nicotine pouches, among total adult respondents from PATH Study Wave 7 (2022-2023)**

|  | **Outcome: Past-30-day use of nicotine pouches** | |
| --- | --- | --- |
|  | **Adjusted Odds Ratio (95% CI)** | **p-value** |
| **Past-12-month receiving discounts or coupons of nicotine pouches** |  |  |
| None | Reference |  |
| Any | **33.85 (19.13, 59.92)** | **<0.001** |
| **Age, years** |  |  |
| 18-24 | Reference |  |
| 25-44 | 0.76 (0.58, 0.99) | 0.041 |
| 45-64 | 0.29 (0.19, 0.42) | <0.001 |
| ≥65 | 0.03 (0.01, 0.12) | <0.001 |
| **Sex** |  |  |
| Female | Reference |  |
| Male | 4.97 (3.42, 7.23) | <0.001 |
| **Race** |  |  |
| White | Reference |  |
| Black | 0.26 (0.13, 0.51) | <0.001 |
| Other | 0.47 (0.29, 0.76) | 0.002 |
| **Ethnicity** |  |  |
| Non-Hispanic | Reference |  |
| Hispanic | 0.37 (0.21, 0.65) | 0.001 |
| **Sexual identity** |  |  |
| Heterosexual | Reference |  |
| Lesbian, gay, bisexual, or other identity | 0.90 (0.61, 1.34) | 0.608 |
| **Annual household income level** |  |  |
| Less than $50,000 | Reference |  |
| $50,000 or more | 1.00 (0.76, 1.33) | 0.983 |

CI=Confidence Interval

**Supplemental Table 1. Results of adjusted modified Poisson regression model (i.e., robust-error-variance Poisson regression model) on past-30-day use of nicotine pouches, among total adult respondents from PATH Study Wave 7 (2022-2023)**

|  | **Outcome: Past-30-day use of nicotine pouches** | |
| --- | --- | --- |
|  | **Adjusted Prevalence Ratio (95% CI)** | **p-value** |
| **Past-12-month receiving discounts or coupons of nicotine pouches** |  |  |
| None | Reference |  |
| Any | 20.73 (14.31, 30.04) | <0.001 |
| **Age, years** |  |  |
| 18-24 | Reference |  |
| 25-44 | 0.77 (0.60, 0.98) | 0.037 |
| 45-64 | 0.30 (0.21, 0.43) | <0.001 |
| ≥65 | 0.03 (0.01, 0.12) | <0.001 |
| **Sex** |  |  |
| Female | Reference |  |
| Male | 4.66 (3.22, 6.74) | <0.001 |
| **Race** |  |  |
| White | Reference |  |
| Black | 0.27 (0.14, 0.53) | <0.001 |
| Other | 0.48 (0.30, 0.77) | 0.002 |
| **Ethnicity** |  |  |
| Non-Hispanic | Reference |  |
| Hispanic | 0.38 (0.22, 0.67) | 0.001 |
| **Sexual identity** |  |  |
| Heterosexual | Reference |  |
| Lesbian, gay, bisexual, or other identity | 0.90 (0.62, 1.31) | 0.595 |
| **Annual household income level** |  |  |
| Less than $50,000 | Reference |  |
| $50,000 or more | 1.00 (0.76, 1.31) | 0.995 |

CI=Confidence Interval

**Supplemental Table 2. Results of adjusted binomial logistic regression model on past-30-day use of nicotine pouches, among total adult respondents from PATH Study Wave 7 (2022-2023), among only young adults (18-24 years old) (N=10,310)**

|  | **Outcome: Past-30-day use of nicotine pouches** | |
| --- | --- | --- |
|  | **Adjusted Odds Ratio (95% CI)** | **p-value** |
| **Past-12-month receiving discounts or coupons of nicotine pouches** |  |  |
| None | Reference |  |
| Any | 21.87 (7.34, 65.14) | <0.001 |
| **Sex** |  |  |
| Female | Reference |  |
| Male | 5.39 (3.39, 8.56) | <0.001 |
| **Race** |  |  |
| White | Reference |  |
| Black | 0.16 (0.03, 0.74) | 0.019 |
| Other | 0.38 (0.21, 0.69) | 0.002 |
| **Ethnicity** |  |  |
| Non-Hispanic | Reference |  |
| Hispanic | 0.27 (0.15, 0.51) | <0.001 |
| **Sexual identity** |  |  |
| Heterosexual | Reference |  |
| Lesbian, gay, bisexual, or other identity | 0.83 (0.49, 1.40) | 0.482 |
| **Annual household income level** |  |  |
| Less than $50,000 | Reference |  |
| $50,000 or more | 1.51 (1.03, 2.23) | 0.037 |

CI=Confidence Interval

**Supplemental Table 3**. **Results of adjusted binomial logistic regression model on past-30-day use of nicotine pouches, among total adult respondents from PATH Study Wave 7 (2022-2023), adjusted for other additional psychological and behavioral factors**

|  | **Outcome: Past-30-day use of nicotine pouches** | |
| --- | --- | --- |
|  | **Adjusted Odds Ratio (95% CI)** | **p-value** |
| **Past-12-month receiving discounts or coupons of nicotine pouches** |  |  |
| None | Reference |  |
| Any | **29.32 (14.35, 59.92)** | **<0.001** |
| **Age, years** |  |  |
| 18-24 | Reference |  |
| 25-44 | 0.78 (0.59, 1.03) | 0.075 |
| 45-64 | 0.37 (0.24, 0.57) | <0.001 |
| ≥65 | 0.06 (0.01, 0.27) | <0.001 |
| **Sex** |  |  |
| Female | Reference |  |
| Male | 3.31 (2.21, 4.93) | <0.001 |
| **Race** |  |  |
| White | Reference |  |
| Black | 0.41 (0.20, 0.85) | 0.017 |
| Other | 0.57 (0.33, 0.99) | 0.045 |
| **Ethnicity** |  |  |
| Non-Hispanic | Reference |  |
| Hispanic | 0.60 (0.34, 1.07) | 0.081 |
| **Sexual identity** |  |  |
| Heterosexual | Reference |  |
| Lesbian, gay, bisexual, or other identity | 0.77 (0.50, 1.19) | 0.238 |
| **Annual household income level** |  |  |
| Less than $50,000 | Reference |  |
| $50,000 or more | 1.46 (1.05, 2.02) | 0.026 |
| **Past-30-day cigarette smoking** |  |  |
| No | Reference |  |
| Yes | 1.11 (0.76, 1.63) | 0.592 |
| **Past-30-day e-cigarette use** |  |  |
| No | Reference |  |
| Yes | 3.39 (2.40, 4.78) | <0.001 |
| **Past-30-day smokeless/snus use** |  |  |
| No | Reference |  |
| Yes | 16.48 (10.95, 24.83) | <0.001 |
| **Past-30-day other substance use** |  |  |
| None | Reference |  |
| Any | 1.14 (0.79, 1.62) | 0.484 |
| **Internalizing tendencies** |  |  |
| Low | Reference |  |
| Moderate | 1.20 (0.81, 1.78) | 0.364 |
| High | 1.15 (0.72, 1.83) | 0.549 |
| **Externalizing tendencies** |  |  |
| Low | Reference |  |
| Moderate | 1.38 (0.95, 2.00) | 0.092 |
| High | 1.43 (0.79, 2.57) | 0.236 |

Other substance use includes alcohol, cannabis, cocaine/crack, misuses of pain killers, sedatives/tranquilizers, stimulants like methamphetamine or speed, heroin, inhalants, or solvents, hallucinogens

CI=Confidence Interval

**Supplemental Table 4**. **Results of adjusted binomial logistic regression model on past-30-day use of nicotine pouches, examining continued use among those who reported ever used nicotine pouches from PATH Study Wave 7 (2022-2023) (N=1,408)**

|  | **Outcome: Past-30-day use of nicotine pouches** | |
| --- | --- | --- |
|  | **Adjusted Odds Ratio (95% CI)** | **p-value** |
| **Past-12-month receiving discounts or coupons of nicotine pouches** |  |  |
| None | Reference |  |
| Any | **5.94 (2.82, 12.51)** | **<0.001** |
| **Age, years** |  |  |
| 18-24 | Reference |  |
| 25-44 | 0.93 (0.69, 1.26) | 0.638 |
| 45-64 | 0.59 (0.37, 0.94) | 0.028 |
| ≥65 | 0.13 (0.03, 0.62) | 0.011 |
| **Sex** |  |  |
| Female | Reference |  |
| Male | 1.47 (0.96, 2.23) | 0.073 |
| **Race** |  |  |
| White | Reference |  |
| Black | 1.09 (0.50, 2.38) | 0.829 |
| Other | 0.84 (0.49, 1.42) | 0.506 |
| **Ethnicity** |  |  |
| Non-Hispanic | Reference |  |
| Hispanic | 1.10 (0.61, 1.98) | 0.754 |
| **Sexual identity** |  |  |
| Heterosexual | Reference |  |
| Lesbian, gay, bisexual, or other identity | 0.86 (0.52, 1.44) | 0.574 |
| **Annual household income level** |  |  |
| Less than $50,000 | Reference |  |
| $50,000 or more | 1.19 (0.89, 1.60) | 0.239 |

CI=Confidence Interval
